# From prediction to explanation for price–volume dynamics in real estate market

**DOI:** 10.1098/rsfs.2025.0069

**Published:** 2026-08-28

**Authors:** Dat Le, Sutharshan Rajasegarar, Wei Luo, Thanh Thi Nguyen, Maia Angelova

**Affiliations:** ^1^ School of Information Technology, Deakin University, Geelong, Victoria, Australia; ^2^ Department of Science, Technology, and International Projects, Ho Chi Minh City University of Economics and Finance, Ho Chi Minh City, 700000, Vietnam; ^3^ Faculty of Information Technology, Monash University, Melbourne, Victoria, Australia; ^4^ School of Science, Technology and Engineering, University of the Sunshine Coast, Sippy Downs, Queensland, Australia; ^5^ Sir Peter Rigby Digital Futures Institute, Aston University, Birmingham, UK

**Keywords:** real estate forecasting, price–volume dynamics, explainable AI, Kolmogorov–Arnold networks, spatio-temporal modelling

## Abstract

Accurate and interpretable real estate forecasting is difficult because housing markets contain heterogeneous price trends, irregular transaction volumes and horizon-dependent price–volume interactions. This study proposes an explainable Kolmogorov–Arnold network (EX-KAN) framework for price forecasting with joint lagged price–volume inputs and competitive transaction-volume prediction. EX-KAN uses lagged price and volume information to forecast future market conditions and applies elasticity-based diagnostics to explain how historical price and transaction volume contribute to predicted prices. Experiments are conducted on an Australian suburban real estate panel with 1826 suburbs and 256 monthly observations from January 2003 to April 2024. EX-KAN is evaluated against KAN, long short-term memory, time-series mixing (TSMixer) and Transformer under a fair joint-input setting across 3-, 6- and 12-month horizons. The results show that EX-KAN achieves the strongest overall price forecasting performance under mean absolute error, mean absolute percentage error (MAPE) and symmetric MAPE, ranking first in six of nine price comparisons and second in the remaining three. Against TSMixer, EX-KAN reduces price MAPE by 17.00% and 8.72% at the three- and six-month horizons, respectively. For transaction-volume forecasting, EX-KAN remains competitive, although KAN is slightly stronger overall. Elasticity, regime-specific and suburb-level analyses show that volume–price relationships vary across horizons, market states and local suburbs.

This article is part of the theme issue ‘Data driven modelling for living systems’.

## 1. Introduction

Forecasting in complex systems, such as financial markets and real estate markets, has long been recognized as a central challenge for both researchers and practitioners [1–3]. Price dynamics are influenced not only by past values of the asset itself but also by trading activity, liquidity flows, macroeconomic factors and behavioural sentiment [4]. These drivers interact in nonlinear and evolving ways, creating dependencies that are often hidden and difficult to disentangle. Accurate and interpretable forecasting of such systems is therefore of great importance for decision-making in investment, policy and risk management, where understanding the underlying mechanisms is as critical as obtaining accurate predictions [5].

Traditional statistical approaches, such as autoregressive integrated moving average (ARIMA) models, have been widely used for forecasting tasks owing to their simplicity, tractability and ease of interpretation [6]. These models perform reasonably well when dynamics are stable and linear assumptions hold. However, they struggle when confronted with structural breaks, sudden shocks and nonlinear cross-dependencies between variables, such as price and volume. Generalized autoregressive conditional heteroscedasticity (GARCH) [7] and related models extend this framework to capture volatility clustering, but they remain fundamentally backward-looking and often fail to adapt to regime shifts triggered by exogenous events, such as financial crises or policy changes.

In recent years, deep learning approaches have emerged as powerful alternatives. Recurrent neural networks (RNNs) [8], long short-term memory (LSTM) models [9] and Transformer-based architectures [10] have demonstrated strong predictive performance in high-dimensional and nonlinear time series. These models excel at capturing complex temporal dependencies and long-range interactions. Nevertheless, they operate as ‘black boxes’, providing little insight into how predictions are generated or how different lagged features contribute to outcomes. This lack of interpretability limits their usefulness in domains such as finance and real estate, where regulators, policymakers and investors require transparency, accountability and explanatory power in addition to raw predictive accuracy.

Explainable artificial intelligence (XAI) [11] methods have been proposed to address this tension. Model-agnostic techniques, such as local interpretable model-agnostic explanations (LIME) [12] and Shapley additive explanations (SHAP) [13], assign importance scores to input variables, but these are *post hoc* approximations and can be unstable. Symbolic regression offers interpretable formulae, but often lacks robustness and scalability. Moreover, most existing XAI approaches for time series focus on input attribution in a static sense, without explicitly modelling how lagged dependencies and cross-variable effects evolve under different market regimes. As a result, the crucial question of how past volume influences future price (and vice versa), particularly across distinct market conditions, remains largely unexplored.

To address this gap, we propose explainable Kolmogorov–Arnold network (EX-KAN), a novel framework that integrates forecasting accuracy with interpretability. Kolmogorov–Arnold networks (KANs) are a recently introduced class of neural architectures inspired by the Kolmogorov–Arnold representation theorem, which guarantees that any multi-variate continuous function can be expressed as compositions of univariate functions and summations. Unlike conventional neural networks that rely on fixed activation functions, KANs learn spline-based functional transformations, offering both universal approximation capability and explicit functional forms that can be inspected after training. This makes KANs uniquely positioned to bridge the gap between predictive performance and interpretability.

Building upon this foundation, EX-KAN introduces three key innovations. First, it uses lagged raw values of both price and volume as inputs, enabling simultaneous forecasting of future price and volume levels rather than modelling returns or anomalies alone. This design makes forecasts more directly interpretable to practitioners who care about actual market levels. Second, EX-KAN computes elasticities from the Jacobians of the trained model, quantifying how changes in past price and volume propagate into future predictions. This provides a principled mechanism to uncover the bidirectional interplay between price and trading activity—something rarely captured by existing forecasting frameworks. Third, EX-KAN incorporates regime-aware analysis by partitioning the time series into high-, normal- and low-price regimes. This allows us to reveal how the strength and direction of price–volume relationships vary across market conditions, providing deeper insight into system behaviour and enabling more context-sensitive decision-making.

Our empirical evaluation, using large-scale panel data of suburb-level real estate prices and transaction volumes, shows that EX-KAN is most effective for short- and medium-horizon price forecasting while maintaining competitive transaction-volume forecasting performance. This asymmetric result is important: the main empirical value of EX-KAN lies in explainable price prediction, whereas transaction-volume forecasting remains noisier and is sometimes better captured by the simpler KAN baseline. Beyond predictive accuracy, EX-KAN provides interpretable lag profiles and regime-specific elasticities that show how historical price and transaction volume contribute to predicted prices across horizons and market states. In particular, the model identifies periods and regimes in which lagged volume is more informative for future price movements, while also showing that this relationship weakens at longer horizons. By combining competitive forecasting with model-based elasticity diagnostics, EX-KAN bridges predictive performance and economic interpretability in a way that is rarely available in standard black-box deep learning models.

The contributions of this paper are threefold:

— We introduce EX-KAN as an explainable price-forecasting framework that uses joint lagged price–volume inputs and also produces competitive transaction-volume forecasts. The model uses Jacobian-based elasticity analysis to quantify how historical price and volume contribute to predicted prices.
— We incorporate regime-aware analysis, showing how price–volume dynamics vary across high, normal and low market conditions, offering a more nuanced understanding of market behaviour.
— We demonstrate through empirical evaluation that EX-KAN achieves forecasting accuracy on a par with state-of-the-art deep learning methods, while providing interpretable and actionable insights absent from black-box approaches.

Through this work, we aim to advance forecasting methodology beyond the trade-off between accuracy and transparency, offering a model that is both performant and interpretable. We argue that EX-KAN represents an important step towards the broader vision of *explainable time-series forecasting*, with potential applications in finance, real estate and other domains characterized by coupled and regime-dependent dynamics.

## 2. Related work

Forecasting housing market dynamics typically requires modelling both prices and market activity (transactions or volume) under non-stationarity, structural breaks and heterogeneous local conditions. Prior work spans econometrics (repeat-sales and hedonic indices, ARIMA/vector autoregression (VAR)/vector error correction model (VECM)) and modern deep learning (RNN/LSTM, Transformer, multi-layer perceptron (MLP)-mixer variants). While these approaches can be accurate, most do not provide transparent, directional explanations of how past price and volume jointly shape future outcomes across market regimes. Our work addresses this gap by pairing competitive forecasts with explicit, regime-aware price–volume elasticities.

### 2.1. Traditional econometric models for housing

Classical time-series models, such as ARIMA and VAR, remain widely used for housing price indices and regional panels owing to their transparency and tractability [6]. Extensions for volatility and structural change (e.g. GARCH-type volatility on returns, regime-switching VAR) help capture clustering and cycles [7]. Hedonic and repeat-sales frameworks are standard for index construction but are often exogenous to short-horizon forecasting. Despite interpretability, linear dependence and stationarity assumptions can limit performance when prices and sales volumes interact nonlinearly, or when cycles (boom–bust, policy shifts and credit tightening) induce regime-dependent dynamics.

### 2.2. Deep learning for real estate forecasting

Deep models handle nonlinearities and long-range dependencies in housing time series and panel data. Recurrent architectures, especially LSTMs, capture temporal memory [9,14]; Transformer-based methods exploit self-attention for long-horizon dependencies [14]; and mixer-style MLPs (e.g. time-series mixing (TSMixer)) improve efficiency on large panels [15], or KANs (e.g. evolving graph convolutional networks) [16]. However, these models are typically black boxes: they provide limited insight into how lagged price and volume jointly influence future levels, making it difficult to interpret drivers of localized market changes, stress episodes or liquidity dry-ups.

### 2.3. Explainable AI for coupled housing dynamics

Model-agnostic XAI (e.g. SHAP and LIME) has been applied to tabular and time-series settings to attribute feature importance [12,13], but explanations are often *post hoc* and local, and may be unstable under perturbations. Symbolic regression offers global forms but can struggle at scale. KANs learn spline-based univariate transformations within a universal approximation framework, yielding tractable functional components [17]. Yet, standard KAN applications seldom quantify bidirectional, regime-sensitive influence between price and volume—an interaction long noted in market microstructure and adapted in housing contexts, where liquidity and search frictions couple trading activity with price adjustment.

### 2.4. Toward interpretable price–volume forecasting in housing

We advance this literature by introducing EX-KAN, which uses lagged raw price and volume to jointly forecast future price (and volume) while computing Jacobian-based elasticities that quantify directional effects of past volume on future price and vice versa. By aggregating these elasticities globally and within data-driven regimes (high-/normal-/low-price states), EX-KAN reveals how coupling strength varies across cycles and stress periods. This design combines the clarity of econometric interpretation (elasticities and regimes) with the expressive power of modern function-learning, addressing a key need in real estate analytics: forecasts that not only are accurate but also explain why and when price–volume linkages intensify or weaken.

## 3. Methodology

This section presents the proposed EX-KAN framework for joint price and transaction-volume forecasting in suburban real estate markets. The method is designed to address two objectives: accurate multi-horizon forecasting and interpretable analysis of price–volume interactions. The full pipeline consists of five stages: (i) price–volume data construction, (ii) lagged supervised learning formulation, (iii) EX-KAN forecasting, (iv) Jacobian-based elasticity analysis, and (v) regime-aware and suburb-level interpretation.

### 3.1. Problem formulation

Let $P_{t,s}$ denote the real estate price of suburb s at month t, and let $V_{t,s}$ denote the corresponding transaction volume. The data form two aligned panel matrices:

(3.1) $$\begin{array}{r} P\in R^{T\times S}\;\text{and}\;V\in R^{T\times S}, \end{array}$$

where T is the number of monthly observations and S is the number of suburbs. In the empirical study, the aligned dataset contains 256 monthly observations and 1826 suburbs.

The objective is to forecast future price and transaction volume at horizon h:

(3.2) $$\begin{array}{r} {\hat{P}}_{t+h,s},{\hat{V}}_{t+h,s}=F_{\theta }\left(P_{t-1:t-L,s},V_{t-1:t-L,s}\right), \end{array}$$

where L is the lag length and h∈{3,6,12} months. In this study, L=6, meaning that each model uses six lagged price values and six lagged volume values.

### 3.2. Lagged price–volume input construction

For each suburb s and time point t, the input vector is constructed by concatenating lagged price and lagged volume values:

(3.3) $$\begin{array}{r} x_{t,s}=\left[P_{t-1,s},\ldots ,P_{t-L,s},V_{t-1,s},\ldots ,V_{t-L,s}\right]. \end{array}$$

The corresponding forecasting targets are future price and future transaction volume:

(3.4) $$\begin{array}{r} y_{t+h,s}=\left[P_{t+h,s},V_{t+h,s}\right]. \end{array}$$

This formulation allows EX-KAN to model within-variable temporal dependence, such as price persistence, and cross-variable dependence, such as the effect of past transaction volume on future price.

To ensure fair benchmarking, the same joint price–volume input structure is also used for all baseline models. Therefore, EX-KAN is not given richer information than KAN, LSTM, TSMixer, or Transformer. Performance differences are therefore attributable to model architecture and learning behaviour rather than unequal input availability.

### 3.3. Explainable Kolmogorov–Arnold network forecasting architecture

EX-KAN extends the KAN principle to joint price–volume forecasting. KANs replace fixed activation functions with learnable univariate transformations, allowing nonlinear relationships to be represented through adaptive basis functions.

A simplified KAN transformation can be written as

(3.5) $$\begin{array}{r} f(x)=\sum_{i=1}^{d}g_{i}(x_{i}), \end{array}$$

where $x\in R^{d}$ is the input vector and each $g_{i}(\cdot )$ is a learnable nonlinear function. In EX-KAN, this idea is used to learn nonlinear relationships among lagged price and volume features.

The EX-KAN forecasting function is defined as

(3.6) $$\begin{array}{r} F_{\theta }:R^{2L}\to R^{2}\;\text{and}\;F_{\theta }(x_{t,s})=\left[{\hat{P}}_{t+h,s},{\hat{V}}_{t+h,s}\right], \end{array}$$

where θ denotes the model parameters. The two output dimensions correspond to future price and future transaction volume.

The key modelling objective is to learn nonlinear price–volume interactions from the lagged input vector:

(3.7) $$\begin{array}{r} \left[P_{t-1:t-L,s},V_{t-1:t-L,s}\right]\to \left[{\hat{P}}_{t+h,s},{\hat{V}}_{t+h,s}\right]. \end{array}$$

Unlike black-box sequence models, EX-KAN retains an explicit functional structure that allows local derivatives with respect to each input feature to be computed after training. This property is central to the explainability component of the framework.

### 3.4. Training objective

EX-KAN is trained by minimizing forecasting error on the training set, with early stopping based on validation performance. The general supervised learning objective is

(3.8) $$\begin{array}{r} L(\theta )=\frac{1}{N}\sum_{\left(t,s\right)}\ell \left(y_{t+h,s},F_{\theta }(x_{t,s})\right), \end{array}$$

where N is the number of training samples and ℓ(⋅) is the forecasting loss. The final evaluation is performed after transforming predictions back to the original price and volume scales.

Because the final experiments evaluate mean absolute error (MAE), mean absolute percentage error (MAPE) and symmetric MAPE (SMAPE), the training set-up is designed to balance absolute and proportional forecasting behaviour. This is important in real estate forecasting because suburbs differ substantially in price levels and transaction frequencies.

### 3.5. Jacobian-based elasticity analysis

The explainability component of EX-KAN is based on the Jacobian matrix of the trained forecasting function [18]. For a given input vector $x_{t,s}$, the Jacobian is defined as

(3.9) $$\begin{array}{r} J(x_{t,s})=\frac{\partial F_{\theta }(x_{t,s})}{\partial x_{t,s}}. \end{array}$$

Since EX-KAN predicts both price and volume, the Jacobian can be written as

(3.10) $$\begin{array}{r} J(x_{t,s})=\left[\begin{array}{cccccc} \frac{\partial {\hat{P}}_{t+h,s}}{\partial P_{t-1,s}} & \cdots & \frac{\partial {\hat{P}}_{t+h,s}}{\partial P_{t-L,s}} & \frac{\partial {\hat{P}}_{t+h,s}}{\partial V_{t-1,s}} & \cdots & \frac{\partial {\hat{P}}_{t+h,s}}{\partial V_{t-L,s}}\\ \frac{\partial {\hat{V}}_{t+h,s}}{\partial P_{t-1,s}} & \cdots & \frac{\partial {\hat{V}}_{t+h,s}}{\partial P_{t-L,s}} & \frac{\partial {\hat{V}}_{t+h,s}}{\partial V_{t-1,s}} & \cdots & \frac{\partial {\hat{V}}_{t+h,s}}{\partial V_{t-L,s}} \end{array}\right]. \end{array}$$

To make these derivatives comparable across variables with different scales, we convert them into scale-adjusted elasticities. For example, the elasticity of predicted price with respect to lagged volume is defined as

(3.11) $$\begin{array}{r} E_{P\leftarrow V_{\ell }}(t,s)=\frac{\partial {\hat{P}}_{t+h,s}}{\partial V_{t-\ell ,s}}\cdot \frac{V_{t-\ell ,s}}{{\hat{P}}_{t+h,s}+\epsilon }. \end{array}$$

Similarly, the elasticity of predicted price with respect to lagged price is

(3.12) $$\begin{array}{r} E_{P\leftarrow P_{\ell }}(t,s)=\frac{\partial {\hat{P}}_{t+h,s}}{\partial P_{t-\ell ,s}}\cdot \frac{P_{t-\ell ,s}}{{\hat{P}}_{t+h,s}+\epsilon }. \end{array}$$

The two main explanatory directions used in this study are

(3.13) $$\begin{array}{r} P\leftarrow P\;\text{and}\;P\leftarrow V. \end{array}$$

The first direction measures the sensitivity of predicted price to historical price. The second direction measures the sensitivity of predicted price to historical transaction volume. These elasticities are summarized using the median, mean, interquartile range (IQR) and positive-share value across test samples.

The positive-share statistic is defined as

(3.14) $$\begin{array}{r} \text{Positive share}=\frac{1}{N}\sum_{i=1}^{N}I\left(E_{i}>0\right), \end{array}$$

where I(⋅) is the indicator function. A positive-share value above 0.5 indicates that the corresponding relationship is positive for most local test samples.

These elasticity values are interpreted as model-based local sensitivities, not causal effects. They explain how the trained EX-KAN model uses lagged price and volume information when producing forecasts.

### 3.6. Regime-aware interpretation

Real estate markets may behave differently under low-, normal- and high-price conditions. To examine whether EX-KAN's price–volume sensitivities change across market states, we conduct regime-specific elasticity analysis.

Each test observation is assigned to one of three regimes based on its price level relative to the empirical distribution:

(3.15) $$\begin{array}{r} \text{Regime}(t,s)=\left\{ \begin{array}{clclc} & \text{Low}, & \; & P_{t,s}\leq q_{0.1}, & \\ & \text{High}, & \; & P_{t,s}\geq q_{0.9}, & \\ & \text{Normal}, & \; & \text{otherwise}, & \end{array}\right. \end{array}$$

where $q_{0.1}$ and $q_{0.9}$ denote the lower and upper quantile thresholds, respectively. Elasticity summaries are then computed separately within each regime.

This regime-aware analysis identifies whether volume-to-price sensitivity is stable across market states or whether the trained model behaves differently in low-, normal- and high-price conditions.

### 3.7. Suburb-level case study analysis

In addition to global and regime-level diagnostics, EX-KAN is examined through selected suburb-level case studies. The purpose is not to establish causal validation, but to provide face-validity checks of local price–volume behaviour.

For each selected suburb, we examine price mean, price standard deviation, volume mean, volume standard deviation and lagged associations between volume growth and future price changes. This local analysis shows whether the global elasticity patterns are consistent with observable heterogeneity across suburbs.

The case-study component is included because real estate markets are spatially and economically heterogeneous. A single global error metric cannot fully describe how model behaviour varies across local markets.

### 3.8. Methodological contributions

The proposed EX-KAN framework contributes to real estate forecasting in four main ways:

— **Joint price–volume forecasting:** EX-KAN uses both lagged price and lagged transaction volume to forecast future price and volume under a unified supervised learning formulation.
— **Fair benchmarking design:** the same joint input structure is used for EX-KAN and all baseline models, ensuring that performance differences reflect model behaviour rather than unequal input information.
— **Elasticity-based explainability:** EX-KAN uses Jacobian-derived elasticities to quantify how lagged price and volume affect predicted price, providing interpretable diagnostics beyond aggregate forecasting metrics.
— **Regime and local interpretation:** EX-KAN supports regime-specific and suburb-level analysis, allowing price–volume relationships to be examined across different market states and local contexts.

Together, these components position EX-KAN as an accurate and interpretable framework for suburban real estate forecasting. The method is designed not only to predict future market conditions but also to explain how historical price and volume information contribute to those predictions.

## 4. Experimental set-up

This section describes the experimental design used to evaluate the proposed EX-KAN framework. The set-up was revised to address the reviewers’ concerns regarding fair benchmarking, repeated-run robustness, statistical testing, ablation analysis and suburb-level interpretation. Figure 1 illustrates the proposed EX-KAN methodology. The framework first aligns monthly price and transaction-volume data, constructs lagged price–volume inputs and then uses EX-KAN to learn nonlinear market interactions for multi-horizon forecasting. The outputs include future price and volume predictions, while model quality and interpretability are assessed through fair benchmarking, accuracy testing, ablation analysis, elasticity diagnostics, regime analysis and suburb-level case studies. All code, configuration files and processed datasets used in this study are available upon reasonable request and released through the project repository: http://github.com/anhdatle/.

**Figure 1.**
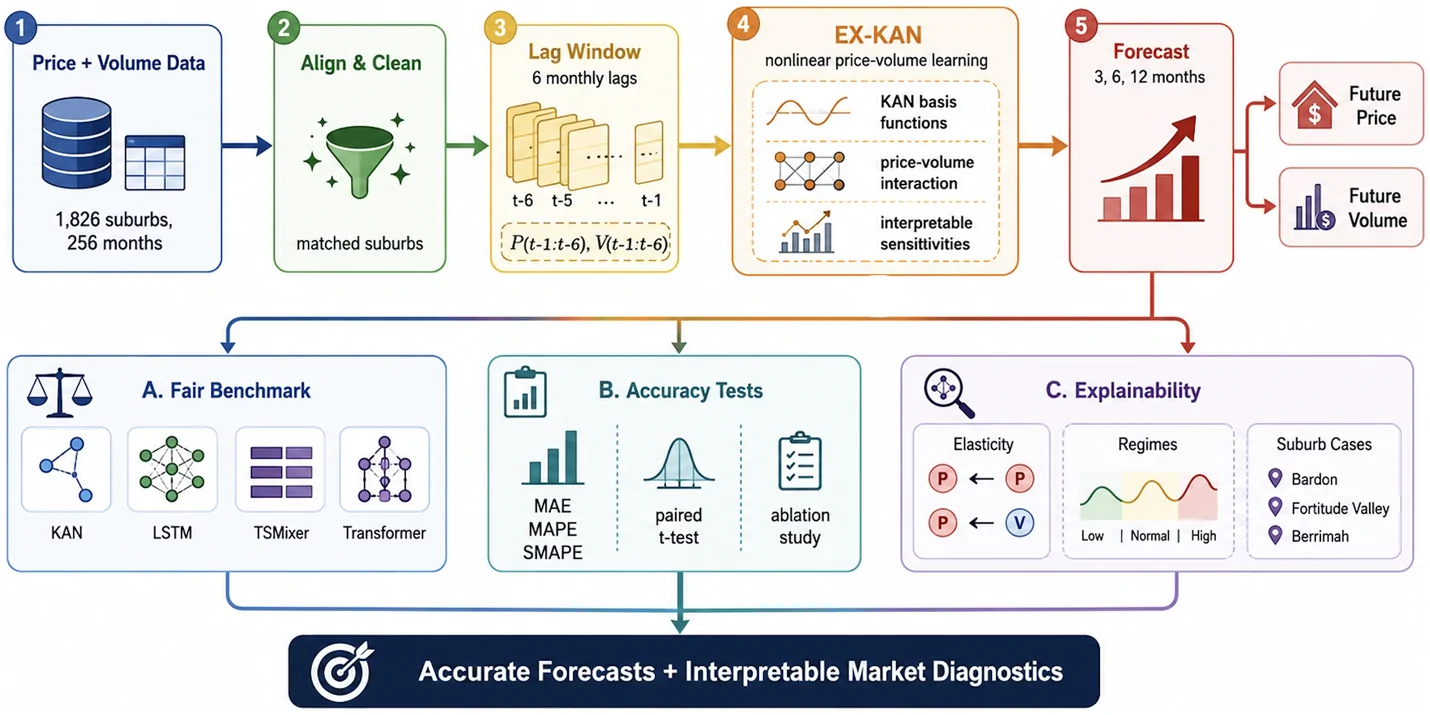
Methodological framework of the proposed EX-KAN model. The pipeline converts aligned price and transaction-volume data into lagged inputs, applies EX-KAN to learn nonlinear price–volume interactions, generates multi-horizon forecasts for price and volume and evaluates the model through benchmarking, statistical testing, ablation analysis and explainability diagnostics.

### 4.1. Dataset

The experiments use monthly real estate panel data consisting of price and transaction-volume observations across Australian suburbs. After cleaning and aligning the two variables, the final dataset contains 1826 suburbs and 256 monthly observations from January 2003 to April 2024. The price matrix and volume matrix have the same structure, where rows represent monthly observations and columns represent suburbs.

Let $P_{t,i}$ denote the price of suburb i at month t, and let $V_{t,i}$ denote the corresponding transaction volume. For each suburb, supervised samples are constructed using lagged price and lagged volume values as predictors. The main forecasting task is evaluated at three horizons: 3, 6 and 12 months.

### 4.2. Input construction

To ensure a fair comparison, all models in the main benchmark use the same joint price–volume input setting. For each target horizon h∈{3,6,12}, the input vector contains six lagged price values and six lagged volume values:

(4.1) $$\begin{array}{r} X_{t,i}=\left[P_{t-1,i},\ldots ,P_{t-6,i},V_{t-1,i},\ldots ,V_{t-6,i}\right]. \end{array}$$

The forecasting targets are future price and future volume:

(4.2) $$\begin{array}{r} Y_{t,i}^{P}=P_{t+h,i}\;\text{and}\;Y_{t,i}^{V}=V_{t+h,i}. \end{array}$$

This joint-input design directly addresses the concern that the proposed model may have benefited from richer input information than the baseline models. Under the revised protocol, all models receive the same lagged price–volume information.

### 4.3. Train–validation–test split

A chronological split is used to avoid lookahead leakage. The first 70% of the time-indexed observations are used for training, the next 10% are used for validation and the final 20% are used for testing. The validation set is used for early stopping and model selection, while the test set is used only for final evaluation.

All input variables and target variables are standardized using statistics estimated from the training set only. The fitted scalers are then applied to the validation and test sets. Final predictions are transformed back to the original scale before computing evaluation metrics.

### 4.4. Compared models

EX-KAN is compared with four baseline models:

— **KAN**: a Kolmogorov–Arnold network baseline using the same lagged price–volume input structure.
— **LSTM**: an RNN baseline designed to model sequential dependencies.
— **TSMixer**: a time-series mixing model that separately mixes temporal and feature dimensions.
— **Transformer**: an attention-based sequence model using the same lagged input window.

The inclusion of KAN evaluates whether the proposed EX-KAN design improves over a simpler KAN-style structure. LSTM and Transformer represent common neural sequence baselines. TSMixer is included as a strong modern time-series baseline.

### 4.5. Training configuration

Each experiment is repeated over five random seeds:

(4.3) $$\begin{array}{r} \{0,7,21,42,66\}. \end{array}$$

The baseline models and EX-KAN are trained for a maximum of 50 epochs with early stopping. Early stopping is based on validation-set performance to reduce overfitting. The batch size is selected based on the dataset size and available Apple Silicon MPS acceleration. Model training uses the AdamW optimizer with weight decay.

All baseline models and EX-KAN were configured using the validation set under the same chronological train–validation–test split. Baseline configurations were selected from preliminary validation experiments and standard architecture settings, while EX-KAN was selected from a small validation-only grid. The test set was never used for hyperparameter tuning or model selection.

### 4.6. Evaluation metrics

The main evaluation uses MAE, MAPE and SMAPE [19]:

(4.4)MAE=1n∑j=1n|yj−y^j|,(4.5)MAPE=100n∑j=1n|yj−y^jyj+ϵ|(4.6) and SMAPE=100n∑j=1n2|yj−y^j||yj|+|y^j|+ϵ.

MAE measures absolute forecasting error in the original target scale. For price forecasting, MAE is measured in price units; for volume forecasting, MAE is measured in transaction counts. MAPE and SMAPE are included because they provide proportional error measures and are more comparable across suburbs with different price levels and transaction frequencies.

RMSE is not used as the main comparison metric because it disproportionately penalizes large absolute deviations and is more sensitive to extreme suburbs. However, RMSE is reported in table S1 in the supplementary material for completeness and comparability with the earlier manuscript version. The RMSE table in the supplementary material follows the same model, horizon and target structure as the main benchmark tables.

### 4.7. Statistical robustness

Statistical robustness is assessed using paired t-tests across the five random seeds. For price forecasting, EX-KAN is compared against TSMixer because TSMixer is the strongest non-EX-KAN price baseline. For volume forecasting, EX-KAN is compared against KAN because KAN is the strongest non-EX-KAN volume baseline. The tests are used as robustness checks rather than definitive evidence of universal superiority, because only five random seeds are available and multiple comparisons are performed across horizons, metrics and targets.

### 4.8. Ablation analysis

An ablation-style analysis is conducted to examine whether EX-KAN improves over the simpler KAN baseline. The ablation comparison reports the relative improvement of EX-KAN over KAN:

(4.7) $$\begin{array}{r} \text{Improvement}=\frac{{\text{Error}}_{\text{KAN}}-{\text{Error}}_{\text{EX-KAN}}}{{\text{Error}}_{\text{KAN}}}\times 100. \end{array}$$

Positive values indicate that EX-KAN achieves lower error than KAN. The ablation analysis is reported separately for price forecasting and volume forecasting, since the two targets have different levels of noise and temporal regularity.

### 4.9. Explainability evaluation

Beyond forecasting accuracy, EX-KAN is evaluated using elasticity-based explainability. Local model sensitivities are converted into scale-adjusted elasticities to quantify how lagged price and lagged volume contribute to predicted price. The main elasticity directions are

(4.8) $$\begin{array}{r} P\leftarrow P\;\text{and}\;P\leftarrow V. \end{array}$$

The first direction measures the sensitivity of predicted price to lagged price. The second direction measures the sensitivity of predicted price to lagged volume. For each horizon, the analysis reports the median elasticity, mean elasticity, IQR and positive-share value. The positive-share value measures the proportion of local sensitivities greater than zero.

These elasticity values are interpreted as model-based sensitivities, not causal effects.

### 4.10. Regime-specific analysis

To examine whether price–volume sensitivity changes across market states, a regime-specific analysis is conducted. Test observations are grouped into low, normal and high regimes. For each regime and horizon, the analysis reports the median volume-to-price elasticity, IQR and positive-share value.

This regime analysis is designed to test whether EX-KAN produces stable explanations across different market conditions. It also addresses the concern that global average elasticity may hide heterogeneous local behaviour.

### 4.11. Suburb-level case studies

Finally, selected suburb-level case studies are used as face-validity checks. The case studies examine Bardon, Fortitude Valley and Berrimah, which represent different price and transaction-volume patterns. For each suburb, the analysis reports price mean, price standard deviation, volume mean, volume standard deviation and lagged volume-growth associations with future price changes.

The case-study analysis is not used as causal validation. Instead, it demonstrates that local price–volume relationships are heterogeneous across suburbs, which motivates the use of elasticity and regime-specific diagnostics in addition to aggregate forecasting metrics.

## 5. Results and discussion

This section reports the revised experimental results of the proposed EX-KAN framework against four baseline models: KAN, LSTM, TSMixer and Transformer. The experiments are designed to address the reviewers’ concerns regarding fair benchmarking, repeated-run robustness, statistical testing, volume forecasting, ablation analysis, elasticity-based interpretation, regime-specific analysis and suburb-level case studies.

All models are evaluated under the same joint-input setting, where each model receives six lagged price variables and six lagged volume variables. Forecasting performance is assessed at three horizons: 3, 6 and 12 months. Each experiment is repeated over five random seeds, and results are reported as mean ± standard deviation. The main comparison focuses on MAE, MAPE and SMAPE. MAE measures average absolute error in the original scale, while MAPE and SMAPE provide proportional error measures that are more comparable across suburbs with different price levels. RMSE is not used as the main comparison metric because it heavily penalizes large absolute deviations and is more sensitive to extreme suburbs.

In the following tables, the best value is shown in **bold**, and the second-best value is shown as *underlined italics*.

### 5.1. Experimental protocol

The dataset contains 1826 suburbs and 256 monthly observations from January 2003 to April 2024. A chronological split is used to avoid lookahead leakage, with 70% of observations used for training, 10% for validation and 20% for testing. The validation set is used for early stopping and model selection. All neural models are trained using standardized inputs and standardized targets, and predictions are transformed back to the original scale before computing the final metrics.

The revised experimental protocol addresses the concern that the original comparison may have favoured EX-KAN through richer input information. In the revised benchmark, all models use the same price–volume input structure. Therefore, the observed differences are attributable to model architecture and learning behaviour rather than unequal input availability.

### 5.2. Price forecasting results

Table 1 reports the price forecasting results. EX-KAN achieves the strongest overall price forecasting performance under MAE, MAPE and SMAPE. At the three- and six-month horizons, EX-KAN outperforms all baseline models across all three metrics. At the 12-month horizon, TSMixer remains slightly stronger, but EX-KAN ranks second and remains close to TSMixer.

**Table 1.** Price forecasting performance using joint price–volume inputs. Values are reported as mean ± standard deviation over five random seeds. Lower values are better. Best values are in bold, and second-best values are underlined and italicized.

horizon (months)	model	MAE	MAPE	SMAPE
3	EX-KAN	**29,386.92±541.01**	4.28±0.05	4.30±0.06
	KAN	40337.74±1933.97	5.46±0.21	5.53±0.24
	LSTM	43553.02±2701.56	5.96±0.32	6.01±0.33
	TSMixer	$\underset{―}{35\;805.89\pm 849.86}$	$\underset{―}{5.15\pm 0.07}$	$\underset{―}{5.16\pm 0.08}$
	Transformer	44187.63±2438.93	6.29±0.43	6.34±0.49
6	EX-KAN	**45,714.55±785.33**	6.61±0.08	6.66±0.10
	KAN	56089.60±1252.09	7.59±0.11	7.68±0.17
	LSTM	56006.41±2050.61	7.93±0.24	8.03±0.29
	TSMixer	$\underset{―}{49\;939.69\pm 663.03}$	$\underset{―}{7.24\pm 0.09}$	$\underset{―}{7.25\pm 0.10}$
	Transformer	55711.14±1163.51	7.99±0.13	8.02±0.08
12	EX-KAN	$\underset{―}{72\;134.73\pm 1,433.93}$	$\underset{―}{10.30\pm 0.21}$	$\underset{―}{10.50\pm 0.21}$
	KAN	77146.28±3975.12	10.70±0.60	11.02±0.78
	LSTM	73639.70±1885.13	10.51±0.16	10.75±0.27
	TSMixer	**70,347.47±942.16**	10.15±0.14	10.33±0.15
	Transformer	79820.62±5482.42	11.27±0.76	11.76±0.95

At the three-month horizon, EX-KAN reduces MAE from 35 805.89 to 29 386.92 compared with the strongest non-EX-KAN baseline, TSMixer. This corresponds to a relative improvement of 17.93%. EX-KAN also reduces MAPE from 5.15 to 4.28% and SMAPE from 5.16 to 4.30%, corresponding to improvements of approximately 17.00 and 16.77%, respectively.

At the six-month horizon, EX-KAN again achieves the best results across all three metrics. Compared with TSMixer, EX-KAN reduces MAE by 8.46%, MAPE by 8.72% and SMAPE by 8.19%. These results indicate that EX-KAN is especially effective for short- and medium-term price forecasting.

At the 12-month horizon, TSMixer achieves the lowest errors, while EX-KAN remains the second-best model. The gap is small: EX-KAN has only 2.54% higher MAE, 1.48% higher MAPE and 1.65% higher SMAPE than TSMixer. EX-KAN still outperforms KAN, LSTM and Transformer at this longer horizon. Overall, across the nine price-forecasting comparisons formed by three horizons and three metrics, EX-KAN ranks first in six comparisons and second in the remaining three.

Although the MAE values appear numerically large, they are measured in the original price scale. Since property prices in the dataset are generally in the hundreds of thousands of dollars, an MAE of 29 386.92 at the three-month horizon corresponds to only 4.28% MAPE. Therefore, MAE should be interpreted together with MAPE and SMAPE rather than in isolation.

### 5.3. Volume forecasting results

Table 2 reports the volume forecasting results. Compared with price forecasting, transaction-volume forecasting is more difficult because volume series are noisier, more irregular and more sensitive to local market activity. The results show that EX-KAN remains highly competitive but does not dominate KAN in volume forecasting.

**Table 2.** Volume forecasting performance using joint price–volume inputs. Values are reported as mean ± standard deviation over five random seeds. Lower values are better. Best values are in bold, and second-best values are underlined and italicized.

horizon (months)	model	MAE	MAPE	SMAPE
3	EX-KAN	$\underset{―}{51.89\pm 0.51}$	$\underset{―}{19.83\pm 0.21}$	16.10±0.11
	KAN	50.31±0.37	19.36±0.32	$\underset{―}{17.58\pm 0.49}$
	LSTM	54.78±0.91	22.20±0.44	18.83±0.37
	TSMixer	58.83±1.43	22.09±0.40	18.81±0.52
	Transformer	78.58±4.50	31.65±2.05	25.26±2.63
6	EX-KAN	$\underset{―}{66.79\pm 0.86}$	$\underset{―}{26.87\pm 0.46}$	$\underset{―}{24.10\pm 0.23}$
	KAN	65.21±0.69	25.64±0.33	23.21±0.29
	LSTM	71.76±0.46	28.68±0.78	24.83±0.26
	TSMixer	76.22±2.11	29.86±0.44	24.46±0.36
	Transformer	93.03±3.17	39.63±1.89	31.37±3.02
12	EX-KAN	$\underset{―}{73.86\pm 0.89}$	$\underset{―}{27.57\pm 0.63}$	$\underset{―}{28.56\pm 0.79}$
	KAN	72.34±0.92	26.77±0.65	27.89±0.67
	LSTM	74.46±1.02	28.02±1.43	28.91±0.31
	TSMixer	81.58±3.49	29.78±1.05	30.43±1.61
	Transformer	79.32±1.61	31.69±1.63	31.36±1.08

At the three-month horizon, EX-KAN achieves the best SMAPE, reducing SMAPE from 17.58 for KAN to 16.10. This corresponds to an 8.45% improvement over the strongest non-EX-KAN baseline on this metric. However, KAN achieves slightly lower MAE and MAPE at the same horizon. The gaps are small: EX-KAN is 3.14% higher in MAE and 2.41% higher in MAPE than KAN.

At the six-month horizon, KAN is the strongest model across all three volume metrics, while EX-KAN ranks second. The difference between EX-KAN and KAN remains moderate: EX-KAN has 2.42% higher MAE, 4.81% higher MAPE and 3.83% higher SMAPE. EX-KAN still clearly outperforms LSTM, TSMixer and Transformer in most metrics.

At the 12-month horizon, KAN again performs best, while EX-KAN remains second across MAE, MAPE and SMAPE. The differences remain small: EX-KAN is 2.09% higher in MAE, 2.97% higher in MAPE and 2.39% higher in SMAPE than KAN. Overall, EX-KAN ranks first or second in all nine volume-forecasting comparisons. Therefore, while KAN remains the strongest volume-specific baseline, EX-KAN provides consistently competitive volume forecasts and avoids the instability observed in LSTM, TSMixer and Transformer.

### 5.4. Overall forecasting comparison

Combining the price and volume results gives a stronger overall picture of EX-KAN. Across the 18 main comparisons, formed by two forecasting targets, three horizons and three metrics, EX-KAN ranks either first or second in every case. For price forecasting, EX-KAN ranks first in six out of nine comparisons and second in the remaining three. For volume forecasting, EX-KAN ranks first in one comparison and second in the remaining eight.

This result shows that EX-KAN provides a stable forecasting framework rather than performing well only under a single target or metric. TSMixer is the strongest competitor for long-horizon price forecasting, while KAN is the strongest competitor for volume forecasting. However, neither baseline is consistently strongest across both targets. By contrast, EX-KAN maintains top-two performance across all selected metrics, targets and horizons.

Compared with LSTM and Transformer, EX-KAN is consistently stronger. In price forecasting, EX-KAN achieves lower MAE, MAPE and SMAPE than both LSTM and Transformer at every horizon. In volume forecasting, EX-KAN also performs better than Transformer across all horizons and remains stronger than LSTM and TSMixer in most comparisons. These results suggest that the proposed KAN-based structure is more suitable for this tabular panel time-series setting than recurrent or attention-based neural baselines.

### 5.5. Statistical robustness

Table 3 shows that EX-KAN achieves statistically clear improvements over TSMixer at the three- and six-month price horizons. At the three-month horizon, paired t-test p-values are below 0.001 for MAE, MAPE and SMAPE. At the six-month horizon, the corresponding p-values remain below 0.01. At the 12-month horizon, TSMixer is slightly better, but the differences are not statistically significant under the paired t-test. This supports the interpretation that EX-KAN is strongest for short- and medium-term price forecasting and remains competitive at the long horizon.

**Table 3.** Paired t-test results for price forecasting. EX-KAN is compared with TSMixer. Positive improvement indicates lower error for EX-KAN.

horizon (months)	metric	EX-KAN	TSMixer	improvement (%)	t-test p
3	MAE	29386.92	35805.89	17.93	<0.001
3	MAPE	4.28	5.15	17.00	<0.001
3	SMAPE	4.30	5.16	16.77	<0.001
6	MAE	45714.55	49939.69	8.46	0.001
6	MAPE	6.61	7.24	8.72	0.001
6	SMAPE	6.66	7.25	8.19	0.001
12	MAE	72134.73	70347.47	−2.54	0.137
12	MAPE	10.30	10.15	−1.48	0.303
12	SMAPE	10.50	10.33	−1.64	0.329

Table 4 shows the statistical comparison for volume forecasting. KAN remains the stronger model in most volume comparisons. EX-KAN achieves the best three-month SMAPE, but KAN has lower MAE and MAPE at the three-month horizon and remains stronger across all three metrics at the 6- and 12-month horizons. These results confirm that EX-KAN's main forecasting advantage is stronger for price than for transaction volume.

**Table 4.** Paired t-test results for volume forecasting. EX-KAN is compared with KAN. Positive improvement indicates lower error for EX-KAN.

horizon (months)	metric	EX-KAN	KAN	improvement (%)	t-test p
3	MAE	51.89	50.31	−3.14	0.004
3	MAPE	19.83	19.36	−2.41	0.073
3	SMAPE	16.10	17.58	8.45	0.002
6	MAE	66.79	65.21	−2.42	0.026
6	MAPE	26.87	25.64	−4.81	0.012
6	SMAPE	24.10	23.21	−3.83	0.039
12	MAE	73.86	72.34	−2.09	<0.001
12	MAPE	27.57	26.77	−2.97	0.016
12	SMAPE	28.56	27.89	−2.39	0.001

Figures 2 and 3 summarize the main experimental evidence for price and volume forecasting, respectively. The results show that EX-KAN achieves the strongest short- and medium-term price forecasting performance, with statistically robust gains over TSMixer at the three- and six-month horizons. For volume forecasting, EX-KAN remains competitive but does not consistently dominate KAN, indicating that its main advantage lies in modelling price dynamics while still maintaining stable performance for transaction-volume prediction.

**Figure 2.**
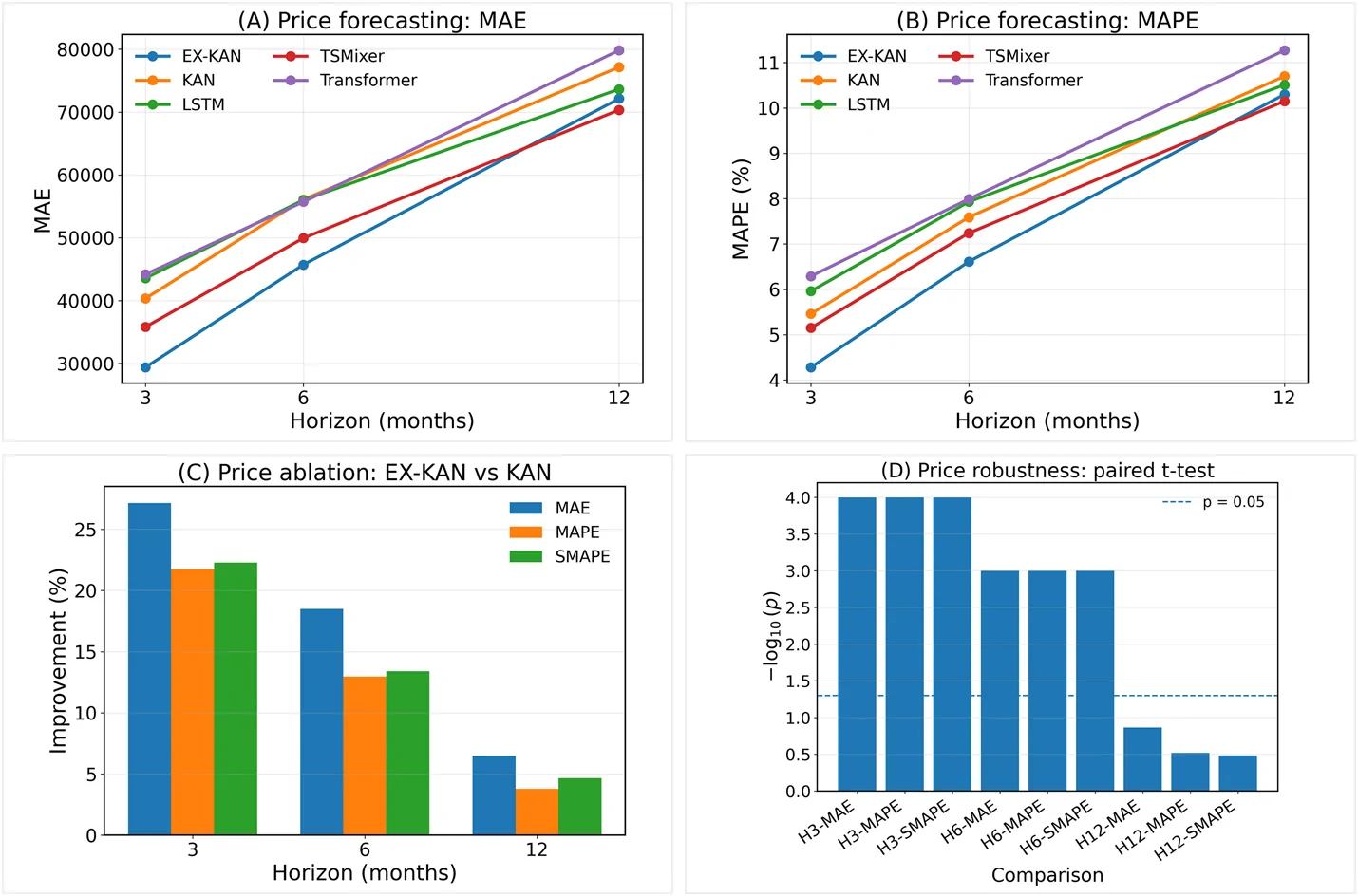
Summary of price forecasting performance, ablation analysis and statistical robustness. (A) Price forecasting MAE across horizons. (B) Price forecasting MAPE across horizons. (C) The relative improvement of EX-KAN over KAN for price forecasting. (D) Paired t-test evidence for EX-KAN against TSMixer in price forecasting, where larger −log⁡(p) values indicate stronger statistical evidence.

**Figure 3.**
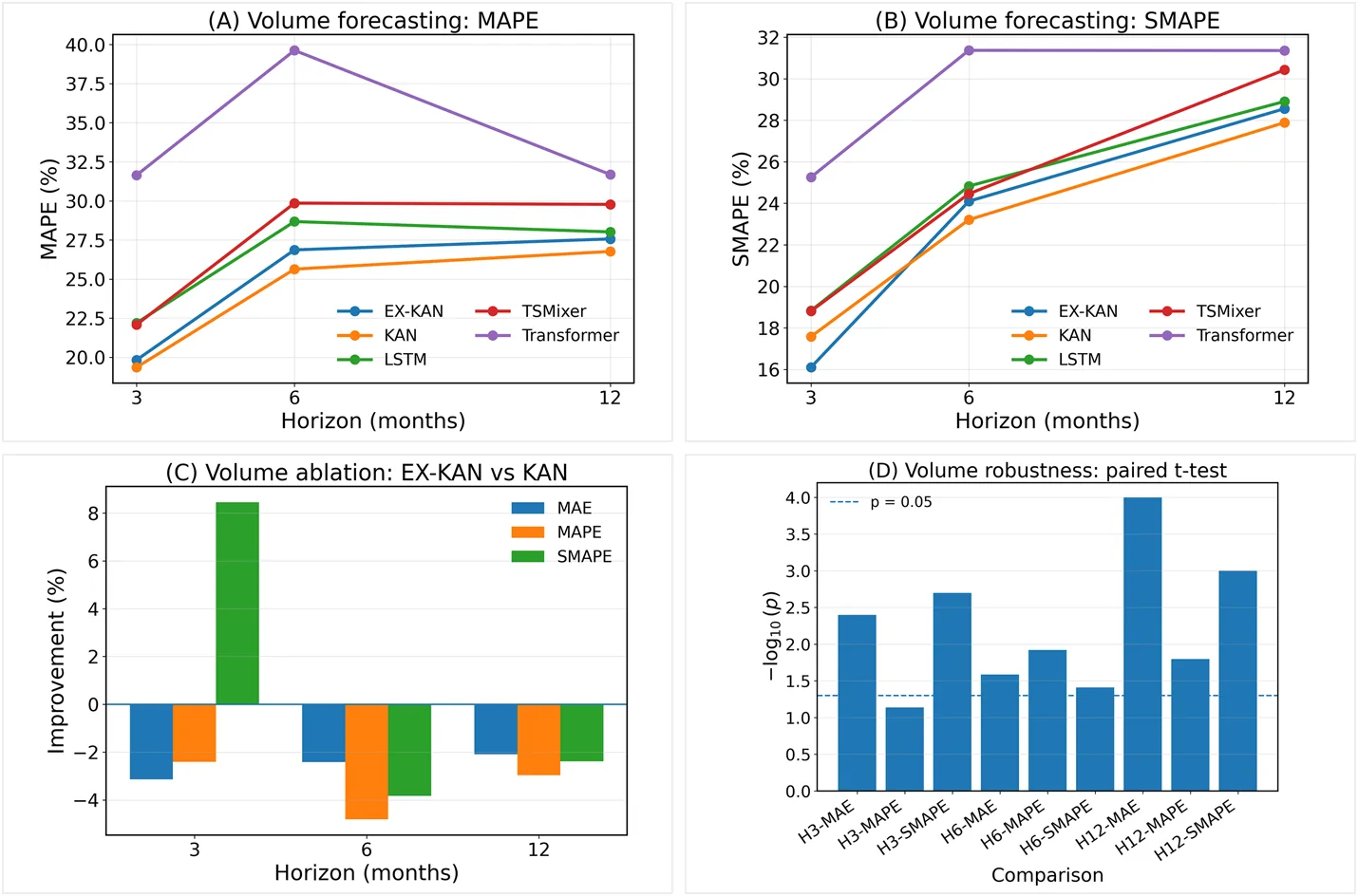
Summary of volume forecasting performance, ablation analysis and statistical robustness. (A) Volume forecasting MAPE across horizons. (B) Volume forecasting SMAPE across horizons. (C) The relative improvement of EX-KAN over KAN for volume forecasting. (D) Paired t-test evidence for EX-KAN against KAN in volume forecasting, where larger −log⁡(p) values indicate stronger statistical evidence.

### 5.6. Ablation analysis

An ablation-style analysis was conducted to examine the contribution of the EX-KAN specification relative to the simpler KAN baseline. Table 5 reports the relative improvement of EX-KAN over KAN. Positive values indicate lower error for EX-KAN.

**Table 5.** Ablation-style comparison of EX-KAN against KAN. Positive values indicate lower error for EX-KAN.

target	horizon (months)	MAE improvement (%)	MAPE improvement (%)	SMAPE improvement (%)
price	3	27.15	21.73	22.27
price	6	18.50	12.96	13.39
price	12	6.50	3.78	4.66
volume	3	−3.14	−2.41	8.45
volume	6	−2.42	−4.81	−3.83
volume	12	−2.09	−2.97	−2.39

The ablation results show that EX-KAN substantially improves over KAN in price forecasting. At the three-month horizon, EX-KAN reduces MAE, MAPE and SMAPE by 27.15%, 21.73% and 22.27%, respectively. At the six-month horizon, the corresponding improvements are 18.50%, 12.96% and 13.39%. At the 12-month horizon, the gains remain positive but smaller, with improvements of 6.50%, 3.78% and 4.66%. These results indicate that the main benefit of EX-KAN is strongest for short- and medium-term price forecasting.

For volume forecasting, the ablation evidence is more conservative. KAN remains slightly stronger in most MAE and MAPE comparisons, while EX-KAN achieves the best three-month SMAPE. This suggests that transaction volume is noisier and less consistently improved by the EX-KAN design than price.

### 5.7. Elasticity-based explainability

Beyond forecasting accuracy, EX-KAN provides elasticity-based explanations of price–volume interactions. The elasticity analysis converts model sensitivities into scale-adjusted measures, allowing the relationship between lagged price, lagged volume and predicted price to be interpreted more directly. These elasticities should be interpreted as local model sensitivities rather than causal effects.

Table 6 summarizes the main elasticity results for price forecasting. The median elasticity of predicted price with respect to lagged price is positive across all horizons. This is expected because real estate prices are persistent over time. The median elasticity of predicted price with respect to lagged volume is also positive at the three- and six-month horizons, with values of 0.0066 and 0.0067, respectively. The positive-share values for the volume-to-price direction are 0.720 and 0.642 at these horizons, meaning that most sampled local sensitivities are positive.

**Table 6.** EX-KAN elasticity summary for price forecasting. Elasticities are scale-adjusted model sensitivities. Positive share denotes the proportion of sampled sensitivities greater than zero.

horizon (months)	direction	median elasticity	mean elasticity	IQR	positive share
3	P←P	0.0093	0.1628	0.1319	0.622
3	P←V	0.0066	0.0006	0.0163	0.720
6	P←P	0.0080	0.1614	0.1813	0.564
6	P←V	0.0067	0.0023	0.0244	0.642
12	P←P	0.0126	0.1929	0.1730	0.594
12	P←V	−0.0030	−0.0019	0.0344	0.452

At the 12-month horizon, the median volume-to-price elasticity becomes slightly negative, and the positive share falls below 0.5. This suggests that lagged volume is more informative for short- and medium-term price forecasting than for long-horizon forecasting. Over longer horizons, the relationship between past transaction activity and future price becomes weaker and less directionally stable.

These results provide an interpretation of the forecasting behaviour. The strong short- and medium-term price forecasting performance of EX-KAN is consistent with the elasticity results, where lagged volume contributes positively to predicted prices at the three- and six-month horizons. At the 12-month horizon, the weaker and less stable volume-to-price elasticity is consistent with the smaller advantage of EX-KAN at longer horizons.

### 5.8. Regime-specific explainability

Table 7 reports regime-specific elasticity diagnostics. The revised regime analysis uses larger regime groups than the original test-window-only formulation, addressing the concern that the high and low regimes previously contained too few observations. The low regime contains 49–58 observations, the normal regime contains 94–109 observations and the high regime contains 333–357 observations, depending on the forecasting horizon.

**Table 7.** Regime-specific EX-KAN elasticity diagnostics. E(P←V) denotes the elasticity of predicted price with respect to lagged volume.

horizon (months)	regime	N	median E(P←V)	IQR	positive share	status
3	low	49	0.0062	0.0200	0.687	descriptive
3	normal	94	0.0102	0.0182	0.798	descriptive
3	high	357	0.0060	0.0158	0.704	descriptive
6	low	52	0.0115	0.0165	0.808	descriptive
6	normal	94	0.0127	0.0210	0.748	descriptive
6	high	354	0.0049	0.0235	0.589	descriptive
12	low	58	0.0042	0.0323	0.624	descriptive
12	normal	109	0.0004	0.0311	0.508	descriptive
12	high	333	−0.0066	0.0363	0.404	descriptive

The regime results show that volume-to-price sensitivity changes across market states. At the three-month horizon, the median P←V elasticity is positive in all regimes, with the strongest value in the normal regime. At the six-month horizon, the low and normal regimes show stronger positive volume sensitivity than the high regime. At the 12-month horizon, the high regime shows a negative median volume-to-price elasticity, suggesting that lagged volume becomes less reliable as a long-horizon price signal in high-price market states.

These findings should be interpreted as descriptive model diagnostics rather than causal evidence. They show how the trained EX-KAN model changes its local sensitivity across market regimes, but they do not prove that volume causally drives future price movements.

Economically, the positive short-horizon P←V elasticity is consistent with liquidity and market-activity effects in housing markets. Higher transaction volume may indicate stronger buyer participation, faster matching between buyers and sellers and more active price discovery, consistent with housing-market search-and-matching models and price–volume models [20,21]. Under this interpretation, recent transaction activity contains useful information for short- and medium-term price adjustment. The weakening of the volume-to-price elasticity at the 12-month horizon suggests that transaction volume is mainly a short- to medium-term signal. Over longer horizons, price movements are likely to be increasingly shaped by broader macroeconomic conditions, affordability constraints, credit availability, interest-rate conditions and local structural factors.

The regime-specific results further suggest that price–volume relationships are not uniform across market states. In low and normal regimes, stronger positive volume-to-price sensitivity may reflect more informative transaction activity and smoother market clearing [21]. In high-price regimes, weaker or negative long-horizon sensitivity may reflect affordability pressure, search frictions and behavioural asymmetries, such as seller loss aversion [20,22]. When prices are already high, additional transaction activity does not necessarily translate into sustained future price growth. These interpretations remain diagnostic rather than causal, but they show that EX-KAN can expose economically meaningful heterogeneity in price–volume relationships across horizons and market regimes.

### 5.9. Suburb-level case study

To provide a concrete interpretation of model behaviour, selected suburbs were examined as suburb-level case studies. This analysis is not used as causal validation. Instead, it provides a face-validity check showing whether observable local price–volume dynamics are consistent with the aggregate elasticity and regime results.

Table 8 summarizes three representative suburbs: Bardon, Fortitude Valley and Berrimah. Bardon has the highest mean price among the selected cases, with a mean price of approximately 710 136.79 and moderate transaction volume. Its same-month price–volume correlation is close to zero, and its lagged volume-to-future-price correlations are weak but mostly positive at longer lags. This suggests that volume contains some short- to medium-term information but should not be interpreted as a strong stand-alone driver.

**Table 8.** Suburb-level case-study summary. The analysis provides face-validity checks rather than causal validation.

suburb	price mean	price std	volume mean	volume std	interpretation
Bardon	710136.79	252708.79	210.36	043.75	weak but mostly positive lagged volume–price associations
Fortitude Valley	455579.35	121935.15	412.59	223.31	volatile transaction activity with mixed lagged associations
Berrimah	539890.49	300380.03	039.18	030.34	smaller and more irregular market with weak negative associations

Fortitude Valley has the highest mean transaction volume and the largest volume standard deviation among the selected cases. Its same-month price–volume correlation is positive but modest. The lagged associations are mixed, indicating that volume spikes do not always translate into proportional future price increases. This supports the need for regime-specific diagnostics rather than a single global interpretation.

Berrimah has the lowest mean volume and a high price standard deviation relative to its market size. Its lagged volume-to-future-price correlations are consistently negative but small. This reflects a smaller and more irregular local market, where transaction volume may behave differently from larger or more liquid suburbs. This case supports the use of both proportional error metrics and local interpretability diagnostics.

The suburb-level cases confirm that the volume–price relationship is heterogeneous across local markets. They do not prove causality, but they support the need for elasticity and regime-specific diagnostics. In some suburbs, volume provides weak positive information for future price changes; in others, the association is mixed or negative. This heterogeneity explains why global average metrics alone are insufficient for interpreting real estate forecasting models.

### 5.10. Discussion

The revised results support four main findings. First, EX-KAN provides the strongest overall price forecasting performance. It outperforms all baselines at the three- and six-month horizons across MAE, MAPE and SMAPE, and remains close to the best model at the 12-month horizon. The statistical tests confirm that the short- and medium-term price improvements over TSMixer are significant under paired t-tests.

Second, EX-KAN remains competitive in volume forecasting, although KAN is slightly stronger overall for this target. EX-KAN ranks first or second in all volume comparisons, but the statistical tests show that its advantage is weaker for volume than for price. This reflects the greater noise and irregularity of transaction-volume series.

Third, the ablation results show that EX-KAN provides substantial gains over KAN in price forecasting, especially at the third- and six-month horizons. The gains are smaller for long-horizon price prediction and weaker for volume forecasting. This suggests that EX-KAN is most effective when recent price–volume interactions remain informative.

Fourth, the elasticity, regime-specific and suburb-level analyses provide interpretability beyond aggregate forecasting errors. Lagged volume contributes positively to predicted price at the three- and six-month horizons, but this relationship weakens at the 12-month horizon and varies across market regimes and suburbs. The case studies further show that local price–volume relationships are heterogeneous, supporting the use of model-based diagnostic tools rather than a single global interpretation.

Overall, EX-KAN offers a strong balance between predictive accuracy and interpretability. It not only is competitive with strong forecasting baselines, but also provides interpretable diagnostics of price–volume interactions. This combination is particularly valuable in real estate forecasting, where users require both accurate predictions and an understanding of how market activity contributes to future price movements.

## 6. Conclusion

This study proposed EX-KAN framework for modelling coupled price and transaction-volume dynamics in suburban real estate markets. Unlike standard forecasting models that mainly optimize predictive accuracy, EX-KAN is designed to combine multi-horizon forecasting with interpretable diagnostics of how lagged price and volume information contribute to future market movements.

The revised experiments show that EX-KAN is most effective for price forecasting, especially at short and medium horizons. Under a fair joint-input setting, where all models use the same lagged price–volume information, EX-KAN achieves the strongest overall price forecasting performance and remains competitive at longer horizons. For volume forecasting, EX-KAN remains stable and competitive, although the simpler KAN baseline performs slightly better in several metrics. This distinction suggests that price dynamics benefit more clearly from EX-KAN's nonlinear price–volume interaction modelling, while transaction-volume forecasting remains a noisier and more difficult task.

Beyond predictive performance, the main contribution of EX-KAN is its ability to provide interpretable market diagnostics. The elasticity and regime analyses show that the influence of volume on future price is not constant across horizons or market states. Volume contributes more clearly to short- and medium-term price prediction, while its effect weakens at longer horizons and varies across local suburbs. These findings support the need for forecasting models that are not only accurate but also able to expose heterogeneous market behaviour.

Overall, EX-KAN provides a practical accuracy–interpretability trade-off for real estate forecasting. It improves price prediction while offering diagnostic tools that help explain when and where price–volume interactions matter. Future work can extend the framework to richer spatial structures, additional macroeconomic variables and other coupled time-series domains, such as financial markets, energy systems, transport demand and regional economic forecasting.

## Data Availability

The datasets analysed in this study consist of suburb-level real estate price and transaction volume data from Australia between 2003 and 2024. All processed data, together with the code required to reproduce the results and figures in this article, are openly available at GitHub: http://github.com/anhdatle/EX-KAN.

## Theme

One contribution of 9 to a theme issue ‘Data driven modelling for living systems’.
